# Biochemometry identifies ostruthin as pluripotent antimicrobial and anthelmintic agent from masterwort

**DOI:** 10.1016/j.isci.2023.107523

**Published:** 2023-08-03

**Authors:** Julia Zwirchmayr, Cristina D. Cruz, Ulrike Grienke, Päivi Tammela, Judith M. Rollinger

**Affiliations:** 1 Department of Pharmaceutical Sciences, Division of Pharmacognosy, Faculty of Life Sciences, University of Vienna, 1090 Vienna, Austria; 2 Drug Research Program, Division of Pharmaceutical Biosciences, Faculty of Pharmacy, University of Helsinki, 00014 Helsinki, Finland

**Keywords:** Medical microbiology, Drugs, Biochemistry

## Abstract

The root extract of *Peucedanum ostruthium* (PO-E) was identified as a promising antibacterial source from a screening of 158 extracts against *Staphylococcus aureus*. It has also recently been shown to significantly decrease the survival of the nematode *Caenorhabditis elegans*. We used the biochemometric approach ELINA to investigate the phytochemical characteristics of the multicomponent mixture PO-E to identify the anti-infective constituent(s) targeting *S. aureus* and *C. elegans.*^1^H NMR spectra of PO-E-derived microfractions were correlated with their respective bioactivity data. Heterocovariance analyses unambiguously identified ostruthin as an anti-staphylococcal constituent, which potently also inhibited *Enterococcus* spp.. ELINA demonstrated that anthelmintic activity was due to a combinatorial effect of ostruthin and isoimperatorin. A *C. elegans*-based survival and motility assay confirmed that isoimperatorin, imperatorin, and verapamil modulated the susceptibility of ostruthin. The combinatorial effect of these natural products was shown in larvae studies to be related to the function of the nematodes’ efflux pump.

## Highlights

• A masterwort extract was identified as an anti-staphylococcal and nematicidal agent
• The biochemometric approach ELINA was applied to unravel bioactive constituents
• ELINA enabled the targeted identification of ostruthin as an anti-staphylococcal agent
• Isoimperatorin adds by efflux-pump inhibition to the nematicidal effect of ostruthin

## Graphical abstract

**Figure:**
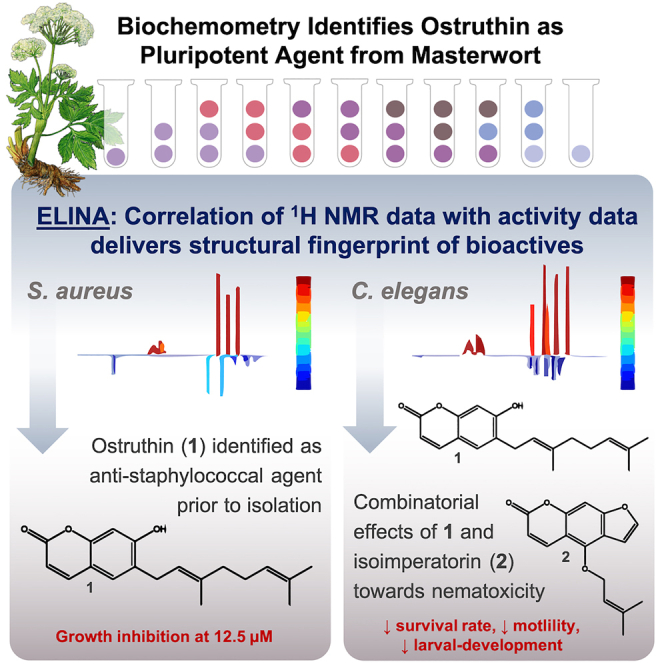

## Introduction

Antimicrobial resistance (AMR) is a major concern in public health care due to therapeutic limitations encountered concerning bacterial infections. Therefore, the search for new antimicrobial agents is crucial. Recent developments in drug discovery show that the number of bacterial species with antibiotic resistance has surpassed the number of new antibiotics introduced.^1^ With the current pace it is predicted that AMR will cause over 10 million deaths per year by 2050 and is the leading threat to human health.^2^ In 2017, the World Health Organization (WHO) released a priority list of drug-resistant microorganisms that pose a major health threat and should be the focus of research and development for new antibiotics. This list was largely comprised of the so-called “ESKAPEE” pathogens—*Enterococcus faecium*, *Staphylococcus aureus*, *Klebsiella pneumoniae*, *Acinetobacter baumannii*, *Pseudomonas aeruginosa*, *Enterobacter* spp., and *Escherichia coli*.^3^^,^^4^ The highest-ranked gram-positive bacteria included in this group are vancomycin-resistant (VR) *E. faecium* and methicillin-resistant *S. aureus* (MRSA).^5^^,^^6^

Intestinal nematode infections are among the most common parasitic infections worldwide and are caused by various species of parasitic worms e.g., by the roundworm *Ascaris lumbricoides* or the hookworm *Necator americanus*.^7^^,^^8^ According to the WHO,^9^ approximately 1.5 billion people (24% of the human population) are infected with one or more helminth species. Although most nematode infections are non-lethal, they often lead to chronic ailments, such as physical disabilities or delayed development of the affected individual. Parasitic nematodes also pose a huge threat to the health of animal livestock and plants, resulting in a serious threat to the global food supply worldwide. Unfortunately, the arsenal of effective anthelmintic agents is limited, and due to the extensive use of anthelmintic compounds in the past, nematode resistance is on the rise.^10^^,^^11^ Currently, there are only a handful of anthelmintic compound classes available (e.g., the macrocyclic lactone ivermectin or the imidazotiazole levamisole) with nematode resistance reported for each class. One reason for this limited number of effective drugs discovered is the complex life cycle of parasitic nematodes. These worms rely on a host for propagation, making it difficult to identify lead compounds with high throughput.^12^ With the increasing prevalence of nematode resistance, however, it is imperative to shorten the time required for the development of effective anthelmintics with less resistance.^13^

The free-living nematode *Caenorhabditis elegans* has emerged as an ideal model organism to study the effects of anthelmintic compounds on e.g., survival, reproduction, and viability *in vivo*.^14^ In the laboratory, *C. elegans* is grown on Petri dishes and fed on the auxotrophic *E. coli* strain OP50. The ease of cultivation for high-throughput screening allows fast identification of both, synthetic^15^ and natural lead compounds in drug discovery.^16^ Its use in whole-organism assays allows for the study of simultaneous interactions between multicomponent mixtures, fractions, and pure compounds against multiple targets in a living organism. In addition, the simplicity and manipulability of nematodes allow for probing large numbers of samples by means of miniaturization and automation,^17^ e.g., semi-automated methods like whole-animal movement.^18^ Although *C. elegans* is not a parasite and lacks many features associated with parasitism, compounds active against *C. elegans* have been reported to be 15 times more likely to kill parasitic nematodes than randomly selected compounds. This highlights the value of *C. elegans* as a pre-screening model for the discovery of anthelmintic compounds.^12^

Nature has proven to be an outstanding source of compounds with manifold therapeutic applications and has been an inspiration for the development of new antibacterial drugs in the past.^19^^,^^20^ According to the comprehensive review by Newman and Cragg,^21^ 162 antibacterial drugs have been approved between 1981 and 2019, of which 11 compounds are natural products (NPs), while 78 are NP-derived (e.g., by semi-synthetic modification). In contrast, the number of antiparasitic compounds is comparably small with only 20 new drugs approved in the same time frame. Of those, only two represent NPs and seven are NP-derived. Six compounds are considered totally synthetic and have been identified via random screening or the modification of an existing drug.^21^ A growing number of research articles aims to identify new anthelmintics from natural sources.^22^^,^^23^^,^^24^^,^^25^^,^^26^ Plant extracts are an important source of new chemical scaffolds with valuable applications for drug discovery.^27^^,^^28^^,^^29^^,^^30^ However, the search for bioactive compounds from complex mixtures (i.e., crude extracts) remains a Herculean task.^31^^,^^32^^,^^33^ Various approaches for the identification and prioritization of a bioactive extract have been described, e.g., knowledge from the ethnomedicinal use of a species in folk medicine followed by a phenotypic screening with a readout that is related to the original use of the species. Nevertheless, the assignment of bioactivity to specific compound(s) is a substantial challenge in NP-based drug discovery.^34^^,^^35^ Bioactivity-guided fractionation approaches are considered the gold standard in NP-based drug discovery. Repetitive fractionation procedures alternate with bioactivity screening and thereby reduce the complexity of the multicomponent mixture successively to isolate the compound(s) responsible for the observed biological effect. A decisive disadvantage is, however, that highly abundant (but potentially inactive) compounds overshadow minor (but potentially active) compounds.^35^ Since no structural information of the actual active compound(s) is given, the most time-consuming step in the bioactivity-guided fractionation approach remains the isolation and identification of the bioactive compound(s). In this light, novel biochemometric approaches for the targeted identification of bioactive compounds have been developed in recent years. Thereby, bioactivity data are correlated with their respective metabolite profiles (e.g., obtained from ^1^H NMR and/or LC-MS measurements) to pinpoint a particular compound to the observed activity.^30^ The biochemometric approach ELINA (Eliciting Nature's Activities) that is employed in this study relies on the deconvolution of a complex mixture (i.e., an active extract or fraction) by generating microfractions with a quantitative variance of constituents over several consecutive fractions.^36^ Correlating bioactivity data with their respective ^1^H NMR data allows for the generation of so-called heterocovariance analysis (HetCA) coefficient plots. Thus, the structural information of compounds contributing to activity can be detected prior to isolation.^37^^,^^38^^,^^39^ By further implementing LC-MS-CAD/ELSD data in the early phytochemical investigation (i.e., after a single fractionation step), ELINA enables early identification of bioactive compounds (e.g., via dereplication and NMR STOCSY analysis) and a target-oriented isolation of the compound(s) of interest.^36^^,^^40^

We rationalized the identification of bioactive NPs through the utilization of a multidisciplinary approach: ethnopharmacological knowledge guided the selection of the starting material and was followed by a subsequent phenotypic-based screening for the identification of antimicrobial starting material. We investigated a total number of 158 small-scale extracts of natural origin for their ability to inhibit the growth of *S. aureus* and *E. coli*. The root extract of *Peucedanum ostruthium* (L.) Koch (PO-E) was revealed as a promising anti-staphylococcal agent. Concurrently, PO-E was shown to significantly reduce the survival rate of *C. elegans*. To accelerate the identification of the bioactive NPs responsible for the observed phenotypic effects on *S. aureus* and *C. elegans*, the biochemometric tool ELINA was implemented in this study *inter alia* for the first time in a whole organism model for anthelmintic screening in *C. elegans*.

## Results and discussion

### Masterwort extract as promising anti-infective against *S. aureus* and *C. elegans*

Within this study, 158 extracts of natural origin (139 herbal and 19 fungal extracts) were tested in a phenotypic-based screening assay at 100 μg/mL on *S. aureus* ATCC 29213 and *E. coli* ATCC 25922 strains (Table S1). The natural materials have been selected based on their reported use in traditional medicine for the treatment of various kinds of infections without in-depth knowledge of their active principle(s). None of the extracts tested at 100 μg/mL showed a significant antibacterial effect on *E. coli* (i.e., ≥50% growth inhibition); however promising results were obtained against *S. aureus*. Three fungal extracts, one extract prepared from the polypore species *Ganoderma applanatum* (Pers.) Pat. (extract 47) and two extracts prepared from different strains of *Piptoporus betulinus* (Bull.) P.Karst (extracts 93 and 94), displayed good antibacterial activity with average inhibition rates of 59%, 52%, and 58%, respectively (Table S1). Two herbal extracts prepared from the roots of *Sophora flavescens* Aiton (extract 124) and *Peucedanum ostruthium* (L.) Koch (extract 150; PO-E), as well as the three rootbark extracts prepared from *Morus alba* L. (extract 156–158), showed excellent inhibitory activities against *S. aureus*, reaching ≥90% growth inhibition (Table S1). Anti-staphylococcal activities for *S. flavescens*^41^^,^^42^^,^^43^ and *M. alba*^44^^,^^45^ have already been reported, whereas the promising activity of PO-E against *S. aureus* has not been described before.

*P. ostruthium,* commonly known as masterwort, has traditionally been used in the Alpine regions of Austria for the preparation of liqueurs, bitters, and teas. The Swiss physician and alchemist Paracelsus (1493–1541 AD) recommended masterwort for the prevention of infections and as a remedy for tuberculosis and worm infections.^46^ The dried and cut roots (i.e., *Radix Imperatoriae*) are valued for alleviating physiological problems including gastrointestinal conditions, and disorders of the respiratory tract and the cardiovascular system.^47^^,^^48^ In a recent study, we could observe that PO-E significantly decreases the survival rate of the nematode *C. elegans* when tested at 100 and 25 μg/mL^17^ and exerts anti-inflammatory activities in a model of IL-1 stimulated endothelial cells.^49^ Based on these results, PO-E was selected for an in-depth investigation focusing on its anti-infective profile against both *S. aureus* and *C. elegans*.

### Target-oriented identification of the active constituent(s) in masterwort against *S. aureus* and *C. elegans* by implementing the ^1^H NMR-based biochemometric approach ELINA

**Figure 1:**
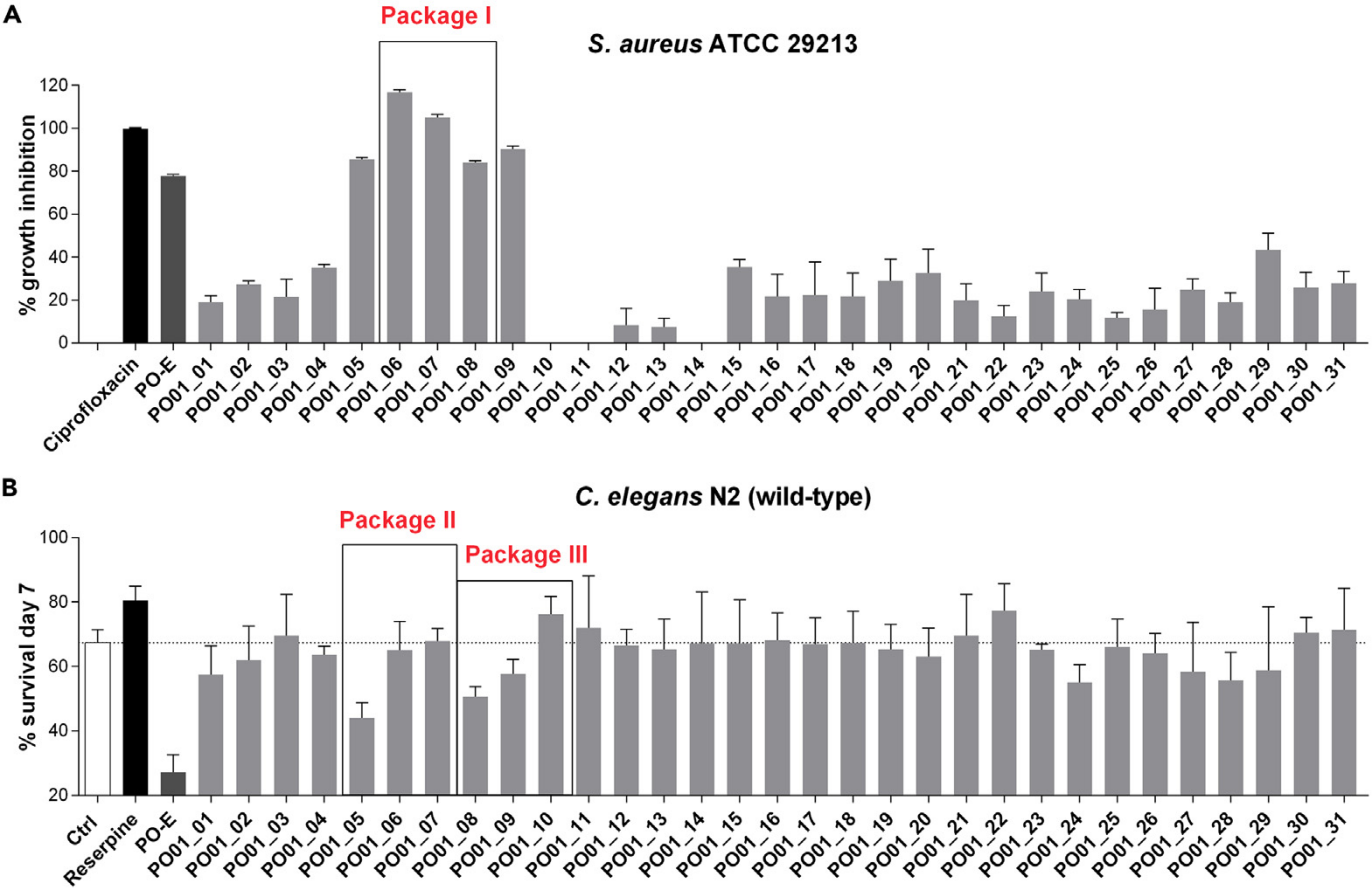
Screening of the 31 PO-E-derived microfractions against *S. aureus* and *C. elegans* (A) *S. aureus* growth inhibition (in percentage ±SD) obtained during the screening of microfractions PO01_01 to PO01_31 (at 100 μg/mL). Data generated from one experiment in triplicate. Ciprofloxacin at 0.5 μg/mL was used as positive control. Package I consist of PO01_06 – PO01_08 (shown as square in panel A; bioactivity data used for the HetCA analysis). (B) *C. elegans* survival rate (in percentage ±SD) at the 7^th^ day of treatment. Nematodes were treated with the vehicle control (DMSO 1%), the positive control reserpine (at 30 μM), PO-E and the 31 microfractions PO01_01 to PO01_31 (tested at 50 μg/mL). Package II consist of PO01_05 – PO01_07, and package III consist of PO01_08 – PO01_10 (shown as square in panel B; bioactivity data used for the HetCA analysis). Data generated from three parallel experiments. Dotted line at 67.35% corresponds to the vehicle control.

The anti-inflammatory potential of *P. ostruthium* was previously investigated using the biochemometric approach ELINA.^40^ For this purpose, ^1^H NMR spectra and LC-MS-CAD data from 31 PO-E-generated microfractions were correlated with their respective bioactivity data from three cell-based *in vitro* assays (i.e., one NF-κB reporter-gene assay and two NF-κB target-gene assays addressing the endothelial adhesion molecules VCAM-1 and E-selectin). By applying this method, several compounds were successfully identified as *in vitro* anti-inflammatory agents prior to isolation. Hence, for the identification of the anti-infective constituent(s) of PO-E, the 31 previously generated microfractions (PO01_01–PO01_31) were probed in the two phenotypic-based screening assays against *S. aureus* and *C. elegans* (Figure 1). Hence, no phytochemical work-up (i.e., microfractionation of PO-E) or analysis (i.e., ^1^H NMR and LC-MS-ELSD measurements) had to be performed as all data necessary for the heterocovariance correlation were already available from the preceding study on masterworts’ anti-inflammatory activities.^40^

The microfractions from PO01_05 to PO01_09 showed the highest percentage of *S. aureus* growth inhibition (i.e., ≥80% at 100 μg/mL). Interestingly, these results were similar to the growth inhibition of PO-E (Table S2; Figure 1A), indicating that the active principle of PO-E against *S. aureus* is concentrated between PO01_05 and PO01_09. MIC values of those microfractions ranged from 6.25 to 100 μg/mL, with the lowest ones achieved by PO01_06 and PO01_07 (i.e., 6.25 μg/mL, Table 1). Strikingly, PO01_05 and PO01_08 (MIC values of 50 and 100 μg/mL, respectively) showed the highest-nematicidal activity in the *C. elegans* survival assay (Figure 1B and Table 1). When tested at 50 μg/mL, both microfractions resulted in an unambiguous reduction of the nematode survival rate of 23% for PO01_05 and 16% for PO01_08. In contrast, the most potent microfractions (i.e., PO01_06 and PO01_07) on *S. aureus* showed no pronounced effect on the survival rate of *C. elegans*, assuming that different compounds/compound classes are contributing to the antimicrobial and nematicidal effects observed in this study.

**Table 1:** Minimum inhibitory concentrations for *S. aureus* ATCC 29213 and *C. elegans* survival data upon treatment with the most active PO-E derived microfractions

Sample	*S. aureus*	*C. elegans*			
	MIC^a^ (μg/mL)	Mean Survival Rate^b^ (%) ± SD	*N*	% Deviation to control	p value
Control	ND	67.35 ± 4.05	259	–	–
Reserpine	ND	80.61 ± 4.31	265	13.26	∗∗
Ciprofloxacin	0.5	ND			
PO-E	50	27.17 ± 5.44	291	−40.18	∗∗∗∗
PO01_05	50	44.02 ± 4.77	101	−23.33	∗∗∗∗
PO01_06	6.25	65.11 ± 8.91	91	−2.24	ns
PO01_07	6.25	50.68 ± 3.13	122	−0.30	ns
PO01_08	100	67.87 ± 3.90	100	−16.67	∗∗
PO01_09	100	57.66 ± 4.53	114	−9.69	ns

a Minimum inhibitory concentrations (μg/mL) are from an average of two assays performed in triplicates. Ciprofloxacin at 0.5 μg/mL was used as positive control.

b *C. elegans*, mean survival rate at the 7^th^ day of treatment is given in percentage ±SD. Worms were treated with the vehicle control (DMSO 1%), reserpine as control (at 30 μM), PO-E and the deriving microfractions PO01_05 – PO01_09 (all at 50 μg/mL). Data generated from three parallel experiments. N is the total number of worms assayed for survival. One way ANOVA with Dunnett’s post-test was used for statistical evaluation. P-value <0.05 was considered statistically significant. ∗∗, p value <0.01, ∗∗∗∗, p value <0.001, ns, not significant.

**Figure 2:**
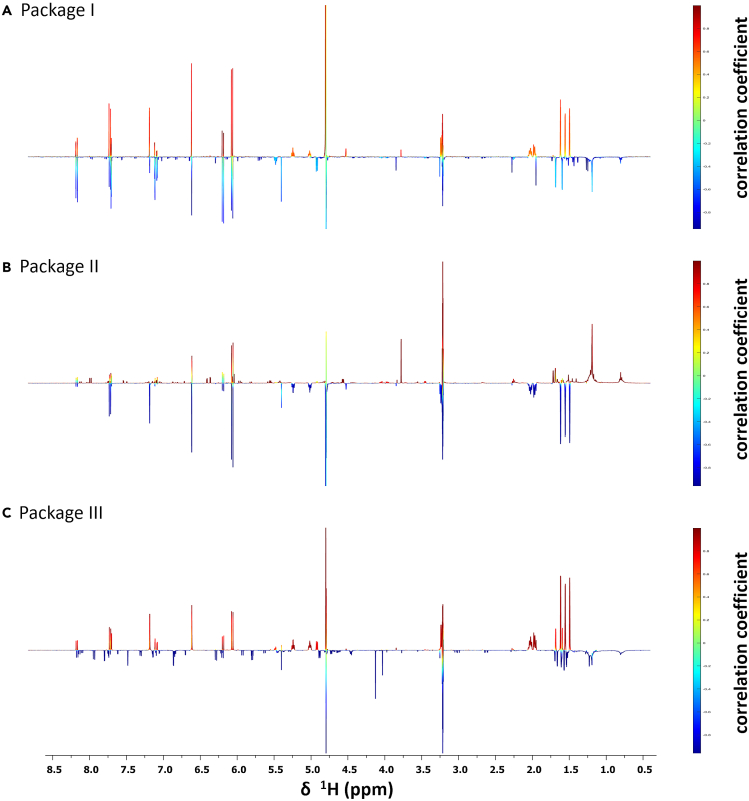
^1^H NMR-based identification of the active principle(s) in PO-E against *S. aureus* and *C. elegans* HetCA Plots of (A) package I, used for the identification of antibacterial agents against *S. aureus*, and (B) package II, and (C) package III, used for the identification of nematicidal agents against *C. elegans*. The color code is based on the correlation coefficient: red signals (“hot features”) are positively, and blue signals (“cold features”) negatively correlated with bioactivity.

For the identification of the anti-infective constituent(s) present within PO-E, packages of consecutive microfractions with increasing/decreasing activities were generated (Figure 1). Package I (consisting of PO01_06–PO01_08) was employed to identify the NPs active against *S. aureus*, whereas package II (consisting of PO01_05–PO01_07), and package III (consisting of PO01_08–PO01_10) were used to identify the nematicidal compounds. By statistically correlating the activity data with their respective ^1^H NMR data, so-called heterocovariance (HetCA) plots were generated (Figure 2) allowing for the identification of structural features from bioactive (but also inactive) constituents within each individual package.^37^^,^^38^ For instance, in Figure 2A typical signals given by fatty acids between *δ*_H_ 1 and 2 are shown, which are defined as “cold features” (blue signals showing downwards), whereas aromatic signals (between *δ*_H_ 6.0 and 8.5) as well as signals given by e.g., methyl groups (between *δ*_H_ 1.5 and 1.8) are displayed as “hot features” (red signals showing upwards). Semi-quantitative LC-MS-ELSD analyses were also performed to visualize the increasing/decreasing peak areas under the curve (AUC) and to facilitate the dereplication of the secondary metabolite(s) present within each package (Figure S1). For instance, the LC-ELSD stack plot of package I indicates the presence of two peaks at an LC retention time (t_R_) of 8.35 and 8.85 min, whereas the AUC of the latter one decreases from the most active microfraction PO01_06 to the least active microfraction PO01_08.

**Figure 3:**
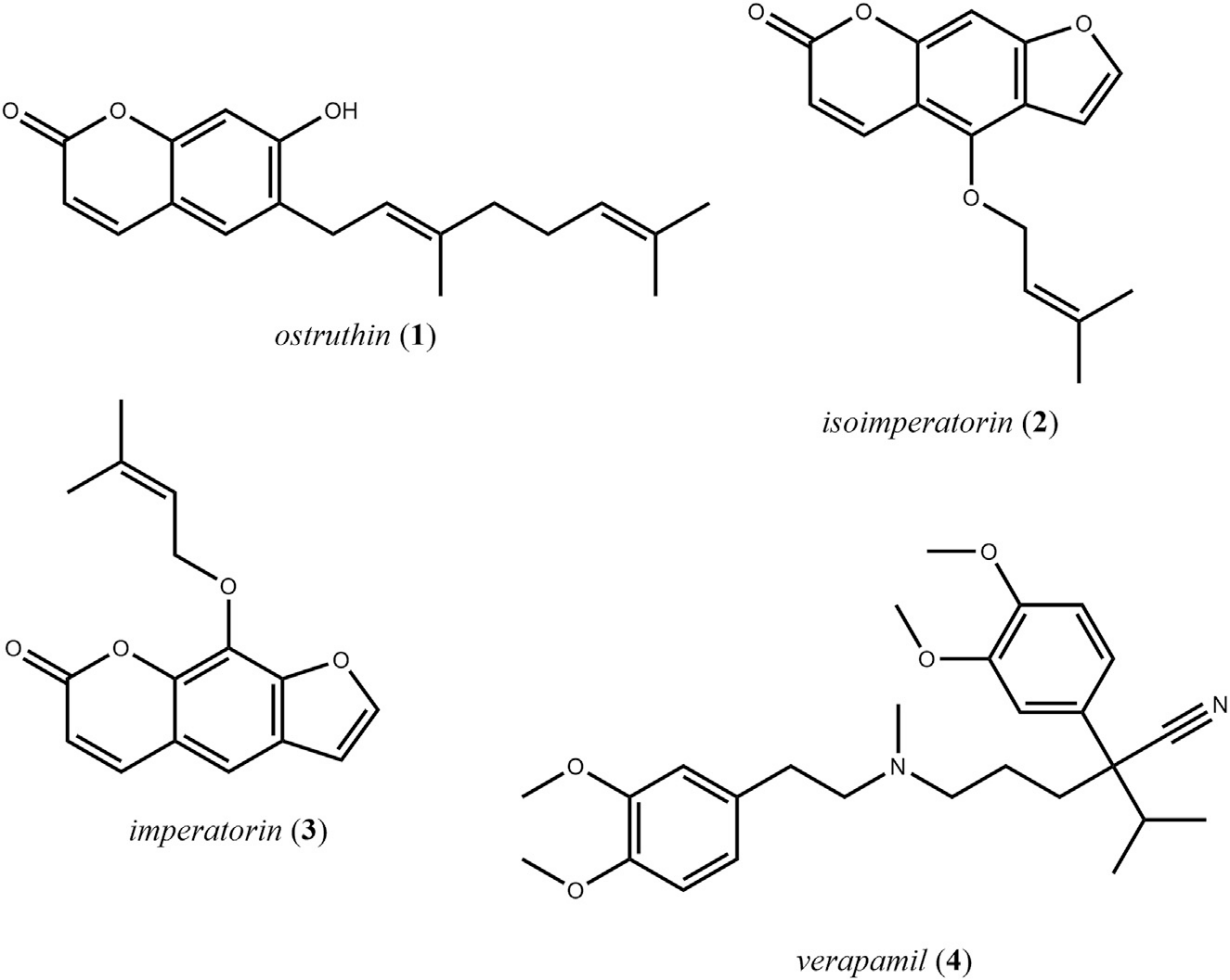
Chemical structures of the compounds investigated within this study The coumarin ostruthin (**1**), the two furanocoumarins isoimperatorin (**2**), and imperatorin (**3**), as well as the Ca^2+^ channel blocker verapamil (**4**).

A dereplication of the selected peak was performed considering an *m/z* value of 299.4 in the positive mode and the structural information given by the generated HetCA plot. Thereby, the coumarin ostruthin (6-geranyl-7-hydroxycoumarin; **1**) was identified as an anti-staphylococcal agent without preceding isolation efforts. The previously isolated coumarin **1**^40^ facilitated the comparison of the positively correlated resonances in the HetCA plot of package I with the ^1^H NMR spectra of **1** (Figure 3). In the upfield chemical shift area, signals at *δ*_H_ 1.59, 1.65, and 1.7 were assigned to the methyl groups at C-3′ and C-7’. The four protons at C-4′ and C-5′ are shown as triplet at *δ*_H_ 2.1 and quartet at *δ*_H_ 2.2, respectively. The two multiplets at *δ*_H_ 5.1 and 5.3 were assigned to the protons at C-2′ and C-6′, respectively. The signal given by the proton at C-1′ is not visible because of an overlay with the solvent signal at *δ*_H_ 3.31. The signals in the downfield chemical shift area assigned to the protons at C-3 and C-4 were observed as two doublets at *δ*_H_ 6.15 and 7.81. Two singlets at *δ*_H_ 6.70 and 6.27 were assigned to the protons at C-8 and C-5, respectively. Thus, the structural predictions delivered by the HetCA plot of package I clearly match the chemical structure of **1**. Likewise, the furanocoumarin isoimperatorin (**2**) with an *m/z* value of 271.1 was identified as the inactive principle in the anti-staphylococcal package I (Figure 3).

ELINA allowed for the fast and target-oriented identification of the anti-infective constituent(s) by applying two assays with different readouts. Although a similar bioactivity range (i.e., in PO01_05 to PO01_09) has been disclosed in both assays, the biochemometric approach identified different chemical features contributing to the observed effects against *S. aureus* and *C. elegans*.

Coumarins are a major compound class in *Peucedanum* species, many of which carry beneficial health properties, such as anti-mycobacterial,^50^^,^^51^ anti-inflammatory,^40^^,^^49^^,^^52^^,^^53^ and antioxidant activities.^54^ Nevertheless, furanocoumarins, a sub-class of coumarins, are known to cause severe phototoxic reactions to humans, such as erythematous rash.^55^ Previously, Vogl et al.^48^ performed a quantification of the main coumarins present in various batches of commercial and field-collected roots of *P. ostruthium*. Compound **1** was identified as the main coumarin present in the dichloromethane extracts of masterwort, with 38–41% of the total coumarin content. In comparison, the content of the furanocoumarins isoimperatorin (**2**) and imperatorin (**3**) was comparably low with 9–10% and 12–15%, respectively.^48^ No studies indicate phototoxic activity of **1**, but some cytotoxic activity has been reported on two pancreatic cell lines.^56^

### Antibacterial effects of ostruthin on *S. aureus* and other gram-positive strains

To further substantiate the biochemometric correlations with antibacterial activity, minimum inhibitory concentrations (MICs) were determined for compounds **1** and **2** against *S. aureus* ATCC 29213 (Table 2). Compound **1** was confirmed to be the solely active principle of package I with an MIC of 12.5 μM. Additional MICs for **1** were determined against MRSA strain ATCC 43300 and four enterococcal strains. Compound **1** has already been reported to possess antibacterial activity against several species of *Mycobacterium*,^50^ however, no information regarding activity against staphylococci or enterococci has been reported so far. Enterococci are the leading cause of health care-associated infections globally, commonly causing urinary tract infections, bacteremia, endocarditis, and wound infections. In humans, *E. faecalis* and *E. faecium* are the most abundant enterococcal species causing about 75% of enterococci-related infections. The intrinsic resistance to many antimicrobials and the capacity to acquire new resistance profiles are major concerns. About 10% of *E. faecalis* isolates and 80% of *E. faecium* isolates are vancomycin-resistant [reviewed by Garcia-Solache and Rice^57^].

**Table 2:** Minimum inhibitory concentrations (μM) of compounds 1 and 2 tested against a panel of gram-positive bacterial strains

Bacterial strains	MIC^a^ in μM (maximum growth inhibition)			
	1	2	Ciprofloxacin	Linezolid
*S. aureus* 29213	12.5	>50 (14.8%)	1.5	ND
MRSA 43300	25	ND	1.5	ND
*E. faecalis* 29212	25	ND	3.0	ND
VR *E. faecalis* 51575	25	ND	1.5	ND
*E. faecium* 35667	25	ND	ND	11.9
VR *E. faecium* 700221	25	ND	ND	11.9

a MIC: Minimum inhibitory concentration was defined as the concentration of a compound that inhibits bacterial growth by ≥ 90%. If the MIC was not achieved, the value is represented by the highest concentration of the compound tested, with respective percentage of maximum growth inhibition in brackets. MR: methicillin resistant; VR: vancomycin resistant; n.d.: not determined. Ciprofloxacin was used positive control for *S. aureus*, MRSA, *E. faecalis* and VR *E. faecalis*. Linezolid was used as positive control for *E. faecium* and VR *E. faecium*. Experiments were performed twice in triplicate.

### ELINA identifies combinatorial effects of 1 and 2 against *C. elegans*

**Figure 4:**
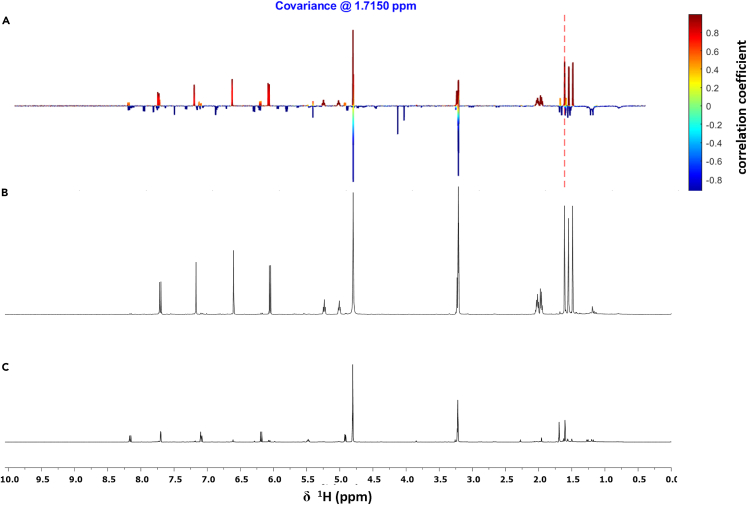
Comparison of the STOCSY plot of package III with the ^1^H NMR spectra of the identified compounds STOCSY plot of package III obtained from the signal at 1.715 ppm (A). The color code is based on the correlation coefficient: red signals (“hot features”) are positively, blue signals (“cold features”) negatively correlated with bioactivity; The stack plot of the ^1^H NMR spectra is depicted for the isolated compounds (B) ostruthin (**1**) and (C) isoimperatorin (**2**).

For the identification of the anthelmintic features in PO-E, the biochemometric approach pinpointed to more than one constituent. As can be seen in package II, the signals of **1** are depicted as “cold features” whereas the signals of **2** are shown as “hot features” (Figure 2B). This is also supported by the increasing AUC of **1** (at t_R_ 8.85 min) from the most active (i.e., PO01_05) to the least active microfraction (i.e., PO01_07; Figure S1B). In package III, on the other hand, the signals of both compounds are displayed as hot features. In Figure 4 the structural predictions delivered by a statistical total correlation spectroscopy (STOCSY) plot are compared to the ^1^H NMR spectra of the isolated compounds **1** and **2**. By applying this method, multiple ^1^H NMR signals from the same molecule can be detected based on their multi-collinearity of their signal intensities in a selected set of ^1^H NMR spectra.^36^^,^^58^ The ^1^H NMR signals of **1** match the dark red signals in the STOCSY plot obtained from the aliphatic signal at 1.715 ppm, whereas the ^1^H NMR signals of **2** match the orange-colored signals in the STOCSY plot. Therefore, the combinatorial effects of **1** and **2** were presumed from the applied omics approach.

**Figure 5:**
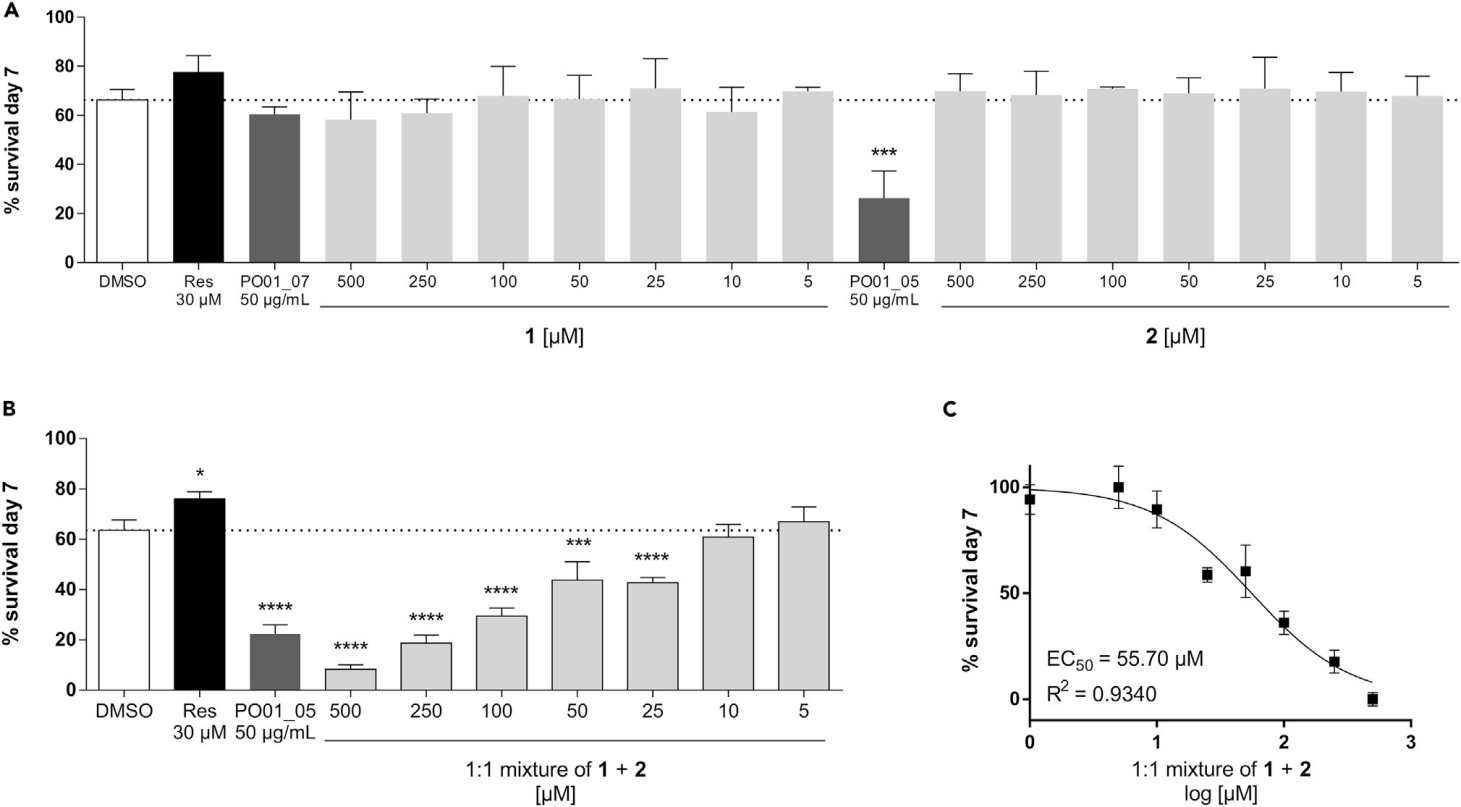
ELINA identifies combinatorial effects of ostruthin (**1**) and isoimperatorin (**2**) against the nematode *C. elegans* (A) *C. elegans* survival rate at the 7^th^ day of treatment. Worms were treated with control, reserpine (30 μM), microfractions PO01_05 and PO01_07 (both at 50 μg/mL) and different concentrations of **1** and **2**, ranging from 500 to 5 μM. Bars represent the mean survival rate (in percentage) on the 7^th^ day of treatment in comparison to the control group ± SD of three parallel experiments. The dotted line at 66.21% corresponds to the vehicle control. (B and C) *C. elegans* survival rate at the 7^th^ day of treatment. Worms were treated with control, reserpine (30 μM), the microfraction PO01_05 (at 50 μg/mL) and **1** and **2** in a 1:1 mixture ranging from 500 to 5 μM. Bars represent the mean survival rate (in percentage) on the 7^th^ day of treatment in comparison to the control group ± SD of three parallel experiments. The dotted line at 63.58% corresponds to the vehicle control. One-way ANOVA with Dunnett’s post-test was used for statistical evaluation. P-value <0.05 was considered as statistically significant. ∗, p value <0.05; ∗∗∗, p value <0.001; ∗∗∗∗, p value <0.0001; (C) Concentration-response curve of **1** and **2** in a 1:1 mixture ranging from 5 to 500 μM. EC_50_ values were determined by non-linear regression with the sigmoidal dose response settings (variable slope) using GraphPad Prism 4.03 software.

To substantiate the assigned nematicidal principle, **1** and **2** were tested at concentrations ranging from 5 to 500 μM in the *C. elegans* survival assay (Figure 5A; Table S3). Intriguingly, when **1** and **2** were tested individually, the survival rate of the worms was not affected. A pronounced and dose-dependent nematicidal response was however observed when **1** and **2** were combined as a 1:1 mixture (Figure 5B), with an EC_50_ of 55.70 μM (Figure 5C).

Driven by the promising nematicidal properties of masterwort and the combinatorial effects of its constituents, we further investigated the impact of these samples on the locomotor activity of *C. elegans*.

### Motility-based screening deciphers ostruthin (1) as main active agent against *C. elegans*

In *C. elegans*, the survival rate depends on two crucial factors: locomotion and feeding. While locomotion represents a strong stimulus for the nematodes to regulate their food intake by increasing their pharyngeal pumping rate, feeding directs their locomotory pattern from dwelling to roaming, depending on the presence of food. Hence, locomotion and feeding behaviors are well coordinated to ensure feeding efficiency.^59^ Loss of motility (i.e., paralysis) is a characteristic phenotypic readout to detect potentially anthelmintic compounds. Among the classes of anthelmintic drugs on the market, macrocyclic lactones, like ivermectin, are the most prevalently used anthelmintics. Macrocyclic lactones act on the glutamate-gated chloride channels, an invertebrate-specific family of ion channels, which are expressed in the nematodes neuromuscular system. Levamisole is also commonly used against nematode infections, acting on specific nicotinic acetylcholine receptors (nAChRs). Both compound classes ultimately induce paralysis of the body wall.^60^^,^^61^

**Figure 6:**
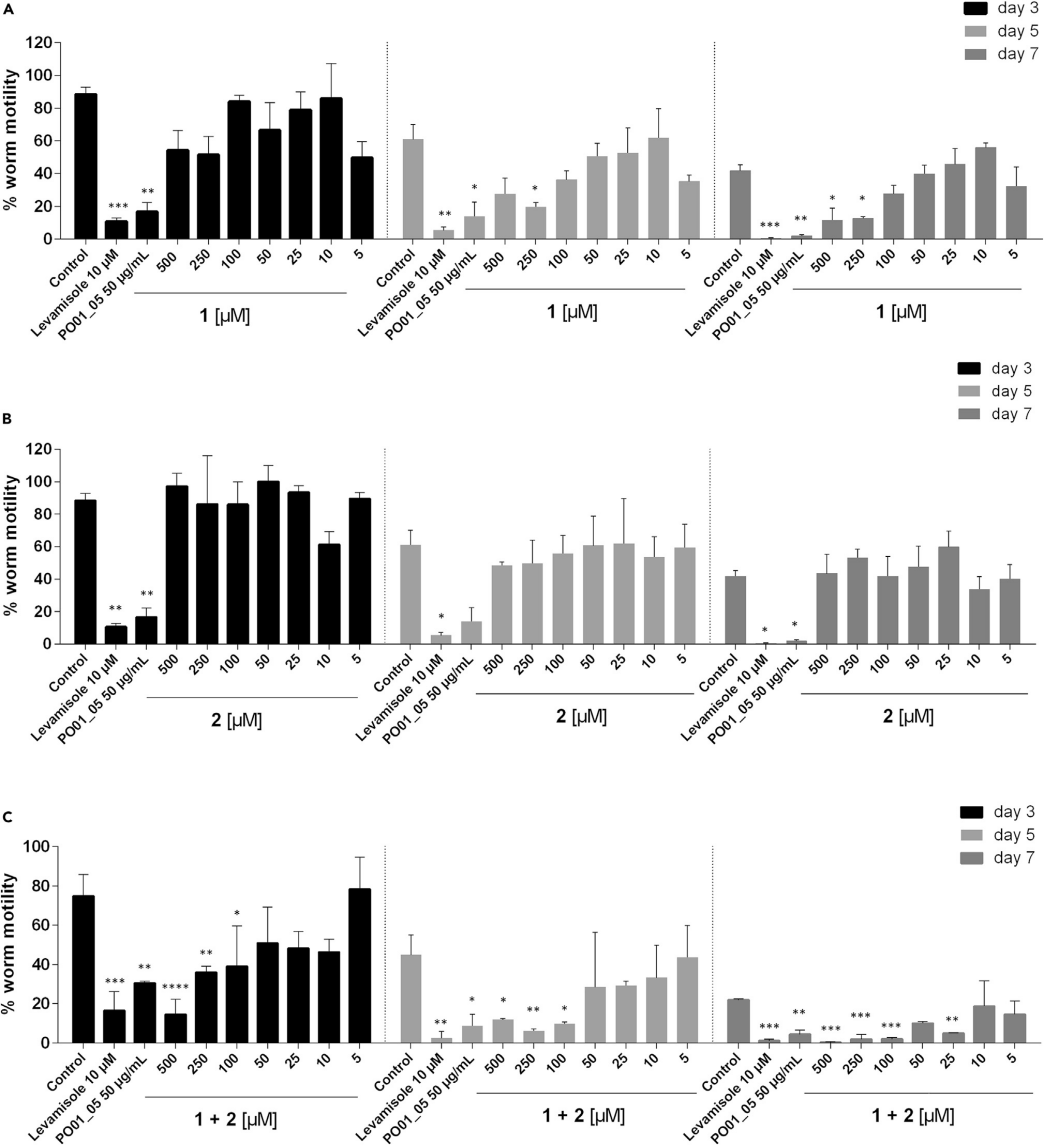
Motility-based screening deciphers ostruthin (**1**) as main active agent against *C. elegans* The effects of **1**, **2** and the 1:1 mixture of **1** + **2** on the motility of *C. elegans* were investigated using an IR-based wormtracker. Worms were treated with control, levamisole (at 10 μM), PO01_05 (at 50 μg/mL) and different concentrations (ranging from 500 μM–5 μM) of (A) the pure compound **1**, (B) compound **2**, and (C) the 1:1 mixture of **1** + **2**. The basal activity of the worms was measured on day 0 and data were normalized to the basal activity. After the worms were treated with the respective samples, their movement was measured on the 3^rd^, 5^th^, and 7^th^ day of the treatment. Bars represent the mean locomotor activity (in percentage) ± SD of three parallel experiments. One-way ANOVA with Dunnett’s post-test was used for statistical evaluation (when compared to the control at the given day). p-value <0.05 was considered as statistically significant. ∗, p value <0.05; ∗∗, p value <0.01; ∗∗∗, p value <0.001; ∗∗∗∗, p value <0.0001.

To examine the effect of **1**, **2**, and their 1:1 mixture on nematodes’ body wall muscles, we employed an automated infrared motility reader in our study (Figure 6; Table S4). This fast and straightforward device provides additional insights into the nematicidal activity^62^ of a sample in the context of a whole-organism-based screening on 96-well microtiter plates. By measuring the locomotor activity of treated vs. control worms, pronounced effects on *C. elegans’* motility were detected for PO01_05 (at 50 μg/mL) with a significant reduction of 81.20% (p < 0.01). In comparison, the levamisole-treated worms (at 10 μM) showed a significant reduction of 88.01% (p < 0.01) on day 3. A dose-dependent reduction of worm motility was shown for the 1:1 mixture of **1** and **2** when tested from 500 to 50 μM on day 3, and from 500 to 5 μM on day 5 and 7, respectively. Strikingly, tendencies of decreasing the nematodes’ motility could also be observed for **1** on the 3^rd^, 5^th^, and 7^th^ day of the experiment. For instance, on the 7^th^ day of the experiment, a dose-dependent reduction of worm motility was observed for compound **1**-treated nematodes with 72.64, 69.79, 34.03, and 4.82% when tested between 500 and 50 μM, respectively. Although the effects were neither as pronounced as for PO01_05, nor as effective as for the 1:1 mixture, these results indicate that **1**, but not **2**, is the main nematicidal principle against *C. elegans*. The furanocoumarin **2**, when tested as a pure compound, showed no effects on the locomotor activity of *C. elegans*.

The findings from the survival and motility assays indicate a modulatory effect of **2** on the susceptibility of **1**. Interestingly, *P. ostruthium* and its isolated compounds have recently been investigated for their anti-mycobacterial properties against *Mycobacterium smegmatis.* Simunovic et al.^51^ investigated *inter alia* **1** and imperatorin (**3**; a constitutional isomer of **2**) regarding their resistance-modulatory effects and efflux pump inhibition in *M. smegmatis.* The authors identified **1** as major antimicrobial compound, whereas the furanocoumarin **3** was found to cause potent modulatory effects via inhibition of the bacterial efflux pump. Compound **3** was not investigated in this study. The reported combinatorial effects of **1** and **2** on the mycobacterial efflux pump as well as the outcome of our nematicidal findings in *C. elegans* prompted us to further investigate a potential interplay of **1** and **2** on the efflux pump of *C. elegans*.

### The modulatory effects of isoimperatorin (2), imperatorin (3), and the efflux pump inhibitor verapamil (4) on the susceptibility of *C. elegans* larvae toward ostruthin (1)

Drug resistance in nematodes can be divided into: (i) specific mechanisms, such as modifications of drug receptors, and (ii) non-specific mechanisms, which are mediated by detoxification enzymes or drug efflux pump pathways. The second group involves multi-drug resistance proteins, such as ATP-binding cassette (ABC) transporters, which attain their protective mechanism through enhanced cellular drug efflux.^63^ Among the ABC transporters, the xenobiotic efflux pump P-glycoprotein (PGP) plays an important part in the sensitivity of nematodes to anthelmintic drugs.^14^^,^^60^^,^^63^^,^^64^^,^^65^^,^^66^ Inhibition of PGPs decreases the efflux of the active drug from the nematode and thus increases the concentration of the drug within the nematode. For instance, the calcium (Ca^2+^) channel blocker verapamil has previously been reported as a PGP inhibitor able to potentiate the efficacy of the anthelmintic drug ivermectin against blood-sucking *Haemonchus contortus* larvae^67^ and *C. elegans* larvae.^14^^,^^64^

**Figure 7:**
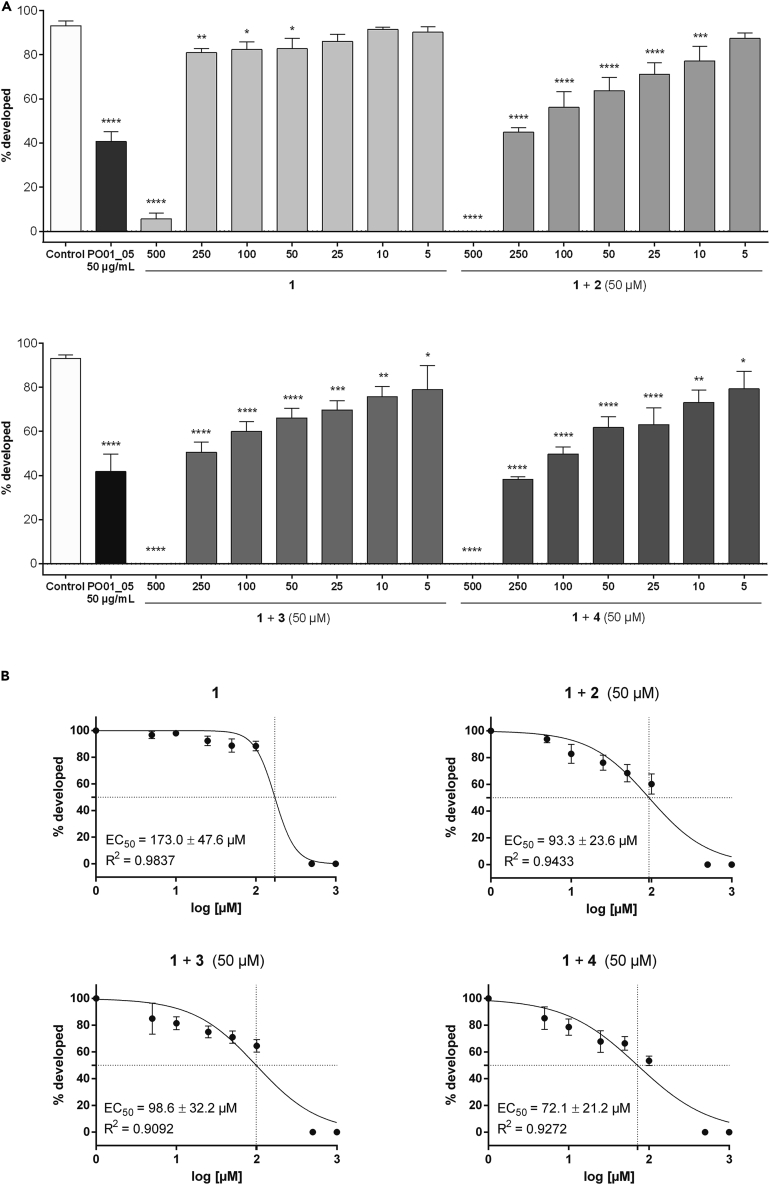
The modulatory effects of isoimperatorin (**2**), imperatorin (**3**) and the efflux pump inhibitor verapamil (**4**) on the susceptibility of *C. elegans* larvae toward ostruthin (**1**) (A) *C. elegans* (N2) L1 larvae were incubated with **1** (at 500 - 5 μM) or co-treatment of **1** (at 500 - 5 μM) in presence of 50 μM **2**, **3**, or **4**, respectively. Percentages of development were calculated as fraction of L1-L3 developmental stages relative to L4 and adult worms. (B) Concentration-response curves of the larval development inhibition assay of *C. elegans* larvae upon treatment with **1** (at 500–5 μM), or co-treatment of **1** in presence of 50 μM **2**, **3**, or **4**, respectively. EC_50_ values were determined by non-linear regression with the sigmoidal dose response settings (variable slope) using GraphPad Prism 4.03 software.

Interestingly, Ca^2+^ antagonistic effects for the furanocoumarins **3** and **2** have previously been reported. The activity of **2**, **3**, and verapamil (**4**) were investigated by measuring the depolarization-induced ^45^Ca^2+^ uptake in GH_4_C_1_ rat pituitary cells, which resulted in IC_50_ values of 6.8, 10.8, and 2.0 μg/mL, respectively.^68^ In another study, the Ca^2+^ antagonistic activity of a *Peucedanum palustre* (L.) Moench root extract was pinpointed *inter alia* to the furanocoumarin **2**.^69^ Consequently, we investigated the effects of the Ca^2+^ channel blockers **2**, **3**, and **4** on the susceptibility of **1**, as well as its impact alone in a larval development-inhibition assay (Figure 7A; Table S5). **1**, when tested alone, impaired the larval development only at the highest concentration tested (i.e., 500 μM) with a larval development inhibition by 93.93%. When tested at concentrations ranging from 250 to 5 μM, the development of *C. elegans* larvae was hardly affected with only 3.2–13.1% deviation to the untreated control. Conversely, all three Ca^2+^ antagonists, when added at 50 μM, dose-dependently increased the susceptibility of **1** toward the larval development, with EC_50_ rates of 93.3, 98.6, and 72.1 μM for **2**, **3**, and **4**, respectively (Figure 7B). Hence, the susceptibility of *C. elegans* larvae toward **1** with an EC_50_ of 173.0 μM was decreased by a factor of 1.854, 1.755, and 2.399 when combined with **2**, **3**, and **4**, respectively. In a previous study, Janssen et al.^14^ reported an increased ivermectin susceptibility of *C. elegans* larvae after inhibition of all PGPs with **4**. The authors reported a 2.5-fold increase in ivermectin susceptibility when worms were co-treated with 50 μM and 100 μM of **4** compared to no co-treatment,^14^ which is in line with our results.

**Figure 8:**
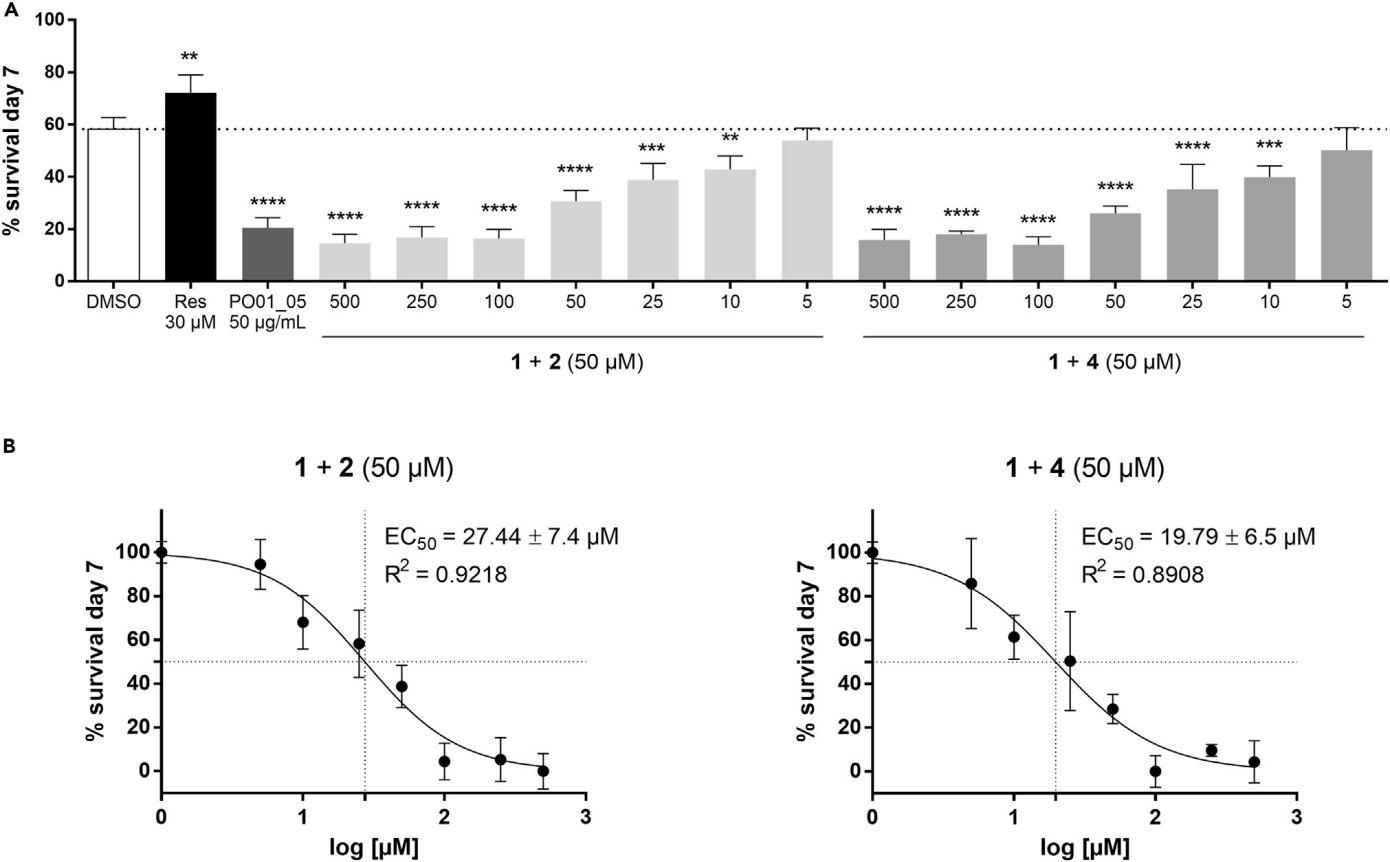
The modulatory effects of isoimperatorin (**2**) and verapamil (**4**) on the susceptibility of ostruthin (**1**) in a *C. elegans* survival assay (A) *C. elegans* survival rate at the 7^th^ day of treatment. Worms were treated with control, reserpine (30 μM), the microfractions PO01_05 (at 50 μg/mL) and different concentrations of **1** (500–5 μM) in the presence of 50 μM **2** or **4**. Bars represent the mean survival rate (in percentage) on the 7^th^ day of treatment in comparison to the control group ± SD of three parallel experiments. Dotted line at 58.21% corresponds to the vehicle control. (B) Concentration-response curves of the *C. elegans* survival assay upon treatment with **1** (500–5 μM), in the presence of 50 μM **2** or **4**. EC_50_ values were determined by non-linear regression with the sigmoidal dose response settings (variable slope) using GraphPad Prism 4.03 software.

EC_50_ calculations from the larval development-inhibition assay were very high, especially when compared to the EC_50_ values obtained from the 1:1 mixture of **1** and **2** in the survival assay with adult worms (Figures 5B and 5C). For a better comparison of the treatments, the survival rate of adult worms was re-evaluated after co-treatment with **1** and 50 μM **2** or **4**, respectively. The modulatory effects of **2** and **4** on the survival rate of adult worms resulted in much lower EC_50_ values than on larval development, with EC_50_ values of 27.44 and 19.79 μM for **2** and **4**, respectively (Figure 8). The reason for the higher efficacy in adult *C. elegans* vs. L1 larvae remains unclear. One explanation could be the differential expression of PGPs in *C. elegans* larvae and adult worms. For instance, Martin et al.^70^ reported a significantly increased expression of the transport protein gene pgp-9 in *Parascaris univalens* larvae exposed to the anthelmintic drugs pyrantel and thiabendazole. On the contrary, drug exposure to adult worms did not significantly increase gene expression.^70^ Nevertheless, the Ca^2+^ channel blocker **2** may act in a similar manner on PGPs as the well-known PGP inhibitor (and Ca^2+^ channel blocker) **4**. However, this is only speculative and warrants further investigations, for example in PGP loss-of-function mutants.

Further, a potential combinatorial effect of **1** and **2** in *S. aureus* was investigated based on previous results reported for imperatorin (**3**).^71^ Compound **2** did not further increase the antibacterial activity of **1** against *S. aureus* when 500 μM of 2 was tested with sub-MIC of **1** (3.13 μM; Table S6). This was in agreement with the biochemometric data. Strain ATCC 29213 also presents no antibiotic resistance linked to efflux. Madeiro et al.^71^ showed synergistic effects of imperatorin (**3**) when tested in combination with erythromycin and norfloxacin (4-fold MIC reduction) on *S. aureus* strain, carrying the gene for overexpression of efflux pumps.

A key benefit of using the whole organism *C. elegans* in the presented ELINA workflow is the nematodes’ simplicity that allows for large numbers of samples to be probed, e.g., in a miniaturized survival assay or by means of a semi-automated motility tracker. Only small amounts of microfractions and their constituents are required. Especially in NP drug discovery, the use of whole-organism assays enables the study of interactions in multicomponent mixtures, fractions, and pure NPs with multiple targets in an organism.^18^ By also implementing a semi-automated motility tracker to the anthelmintic screening, the nematicidal **1** was detected as well as the modulatory effect of **2** could be disclosed. The involvement of the nematodes’ efflux pump was investigated and substantiated the modulatory effects of the known efflux pump inhibitors **2**, **3**, and **4** on the susceptibility of **1** in a larval development-inhibition assay. In sum, the implementation of *C. elegans* as a holistic multipurpose tool for bioactivity studies together with the target-oriented biochemometric ELINA approach, aids the exploration of pleiotropic molecular mechanisms *in vivo*. To our knowledge, this is the first time where a dual antibacterial and nematicidal activity is described for the coumarin ostruthin.

### Limitations of the study

The study demonstrated the nematicidal properties of PO-E in *C. elegans*. However, none of the tested PO-derived microfractions showed similarly strong effects toward the nematodes’ survival rate as the complex multi-component mixture PO-E. Therefore, it is likely that further NPs with nematicidal properties are present in PO-E potentiating the observed effects of **1** and **2**. The results from the non-parasitic *C. elegans* model are also not completely transferable to any parasitic nematode, and this warrants further investigation in parasitic nematodes species to substantiate the anthelmintic potential of PO-E.

## STAR★Methods

### Key resources table

**Table:** 

REAGENT or RESOURCE	SOURCE	IDENTIFIER
**Bacterial and virus strains**		

*Escherichia coli* OP50	CGC	https://cgc.umn.edu/
*Escherichia coli*	American Type Culture Collection	ATCC 25922
*Staphylococcus aureus*	American Type Culture Collection	ATCC 29213
Methicillin-resistant *Staphylococcus aureus*	American Type Culture Collection	ATCC 433000
*Enterococcus faecalis*	American Type Culture Collection	ATCC 29212
Vancomycin-resistant *Enterococcus faecalis*	American Type Culture Collection	ATCC 51575
*Enterococcus faecium*	American Type Culture Collection	ATCC 35667
Vancomycin-resistant *Enterococcus faecium*	American Type Culture Collection	ATCC 700221

**Chemicals, peptides, and recombinant proteins**		

(±)-verapamil HCL	Sigma-Aldrich	V4629
Agar-Agar	Carl Roth	6494.3
CaCl_2_	Merck	1.02378
Cation adjusted Mueller Hinton Broth	BD Biosciences	212322
Cholesterol	Sigma-Aldrich	C8503
Ciprofloxacin hydrochloride	ICN Biomedical Inc.	Cat# 199020
Citric acid monohydrate	Sigma-Aldrich	C1909
CuSO_4_ x 5 H_2_O	Sigma-Aldrich	C8027
Dimethyl sulfoxide (DMSO)	VWR Chemicals	23500.297
FeSO_4_ x 7 H_2_O	Sigma-Aldrich	215422
FUdR (5-Fluoro-2‘-deoxyuridine)	Sigma-Aldrich	F0503
K_2_HPO_4_	Sigma-Aldrich	P3786
KH_2_PO_4_	Sigma-Aldrich	P5655
Levamisole HCL	Sigma-Aldrich	L9756
Linezolid	Sigma-Aldrich	PZ0014
MgSO_4_	Sigma-Aldrich	M7506
MnCl_2_ x 4 H_2_O	Sigma-Aldrich	63535
Mueller Hinton Agar	Neogen	NCM2016A
Na_2_EDTA	Sigma-Aldrich	E1644
Na_2_HPO_4_ x 2 H_2_O	Sigma-Aldrich	S7907
NaCl	Sigma-Aldrich	S7653
NaClO	Sigma-Aldrich	1.05614
NaOH	Sigma-Aldrich	S8045
Peptone from meat	Sigma-Aldrich	91249
Reserpine	Sigma-Aldrich	83580
Tri-potassium citrate monohydrate	Sigma-Aldrich	60153
Yeast Extract	Sigma-Aldrich	Y1625
ZnSO_4_ x 7 H_2_O	Sigma-Aldrich	Z4750

**Experimental models: Organisms/strains**		

*Caenorhabditis elegans* Strain N2 (wild-type)	Caenorhabditis Genetics Center (CGC)	https://cgc.umn.edu/

**Software and algorithms**		

Excel	Microsoft	https://www.microsoft.com/
MestReNova	Mestrelab Research	https://mestrelab.com/
Matlab	The MathWorks, Inc.	https://de.mathworks.com/
GraphPad PRISM	Graphpad Software, Inc.	https://www.graphpad.com/

### Resource availability

#### Lead contact

Further information and requests for resources and reagents should be directed to and will be fulfilled by the lead contact, Prof. Dr. Judith M. Rollinger (judith.rollinger@univie.ac.at).

#### Materials availability

This study did not generate new unique reagents.

### Experimental model and study participant details

#### Extracts

Most of the herbal and fungal materials investigated within this study have been part of previous publications, i.e., extract 1–152 (see Table S1). For most of the extracts, a protocol for the enrichment of constituents suitable for high-throughput screening techniques was applied (i.e., lead-like enhanced extracts, LLE). All LLE extracts were prepared according to previous protocols,^17^^,^^49^^,^^72^^,^^73^ adapted from Camp et al.^74^ Detailed information about the source and the extraction procedures have already been reported for extract 1–143,^72^ extract 145–149,^17^ extract 150,^40^ extract 151,^75^ extract 152,^76^ extract 153–155,^77^ extract 156^78^ and extract 157–158.^79^ Voucher specimens are deposited in the Herbarium of the Division of Pharmacognosy, Department of Pharmaceutical Sciences at the University of Vienna, Austria. Extracts were either generated according to the protocol of lead-like enhanced (LLE) extraction as described earlier,^17^^,^^73^^,^^74^ with ethanol (E), dichloromethane (D, CH_2_Cl_2_), with methanol (M, CH_3_OH), accelerated solvent extraction (ASE) or supercritical-fluid extraction (SFE).

#### PO-E

Dried *Peucedanum ostruthium* roots and rhizomes were purchased from Kottas Pharma GmbH (Ch.Nr.: P17301770), Vienna. A voucher specimen (JR-20180119-A2) is deposited at the Department of Pharmaceutical Sciences, Division of Pharmacognosy, University of Vienna, Austria. The extraction procedure and the generation of the microfractions has already been described.^40^ Briefly, 1 kg of dried plant material was defatted with *n*-hexane and subsequently extracted with CH_2_Cl_2_ and CH_3_OH. CH_2_Cl_2_/CH_3_OH extracts were combined and concentrated to dryness on a rotary evaporator. The extraction yielded 348.96 g extract (i.e., PO-E; 35.81%). Microfractionation of PO-E was performed via HPCCC in a semi-preparative, normal-phase mode with gradient elution (starting with HEMWat system 22 and gradually increasing the polarity of the mobile phases by subsequently by applying the mobile phases (i.e., upper layer) of HEMWat system 21, 20, 19, 17, 15, and 10). Two runs were performed in total and 565 HPCCC fractions collected. All HPCCC fractions were monitored by TLC and pooled to obtain 31 final microfractions, i.e., PO01_01–PO01_31. An aliquot of the microfractions was dissolved in DMSO (Rotipuran ≥99.8%, p.a.) to a final concentration of 10 mg/mL and stored at −20°C until further use.

#### Bacterial strains and growth conditions

Bacterial strains were purchased from Microbiologics Inc. (St. Cloud, MN, USA), and reconstituted according to manufacturer’s instructions. Bacterial stocks were prepared in cation-adjusted Mueller Hinton broth (CAMHB, BD) and stored at −80°C. Fresh cultures were initiated monthly on Mueller Hinton agar (MHA, BD) plates. Overnight cultures were prepared before the assay by subculturing bacterial strains on fresh MHA plates and incubated at 37°C for 16–20 h. The following bacterial reference strains were used in this study: *Escherichia coli* ATCC 25922; *Staphylococcus aureus* ATCC 29213; MRSA ATCC 43300; *Enterococcus faecalis* ATCC *29212, VR E. faecalis ATCC* 51575, *Enterococcus faecium* ATCC 35667 and VR *E. faecium* ATCC 70021.

#### *Caenorhabditis elegans* strain and maintenance

*Caenorhabditis elegans* wild-type var. Bristol N2 and uracil auxotroph *Escherichia coli* OP50 were obtained from the *Caenorhabditis* Genetics Center (University of Minnesota). OP50 were grown in Luria-Bertani (LB) medium (NaCl, peptone, yeast extract, Sigma-Aldrich) for 8 h at 37°C, harvested through centrifugation and washed with double distilled water. Then, bacteria were suspended in S-complete medium (Cholesterol, citric acid monohydrate, CuSO_4_, FeSO_4_, K_2_HPO_4_, KH_2_PO_4_, MgSO_4_, MnCl_2_, Na_2_EDTA, NaCl, tri-potassium citrate monohydrate, ZnSO_4_, Sigma-Aldrich; CaCl_2_, Merck) at a concentration of 100 mg/mL. Flasks were stored at 4°C until use. Hermaphrodite animals were maintained on nematode growth medium (NGM; Agar-Agar, cholesterol, K_2_HPO_4_, KH_2_PO_4_, NaCl, peptone, Sigma-Aldrich; CaCl_2_, Merck) agar plates seeded with 200 μL of OP50 solution at 16°C according to standard protocols.^80^ For maintenance, worms were transferred to new NGM plates every week and cultures were monitored on a regular basis. For the preparation of a synchronized worm populations, N2 worms were chunked three days before synchronization. Synchronization was performed by bleaching technique.^81^^,^^82^ Briefly, worms were washed off the plates with double distilled water and treated with bleaching solution (NaClO, NaOH) for approximately 5–7 min. The lysis of the worms was controlled under a dissecting microscope (Z1 Axio Observer, Zeiss). Isolated eggs were then pelleted and washed twice with M9 buffer (K_2_HPO_4_, KH_2_PO_4_, MgSO_4,_ NaCl, Sigma-Aldrich) and S-complete medium. Eggs were kept in S-complete medium for 42 h with gentle agitation and sufficient aeration until the synchronized population of nematodes hatched.

### Method details

#### Antibacterial screening assays and minimum inhibitory concentration (MIC) determinations

A primary screening of 158 herbal/fungal extracts was performed to investigate inhibition of growth of *E. coli* ATCC 25922 and *S. aureus* ATCC 29213. Antimicrobial susceptibility tests were based on the broth micro-dilution method according guidelines by the Clinical and Laboratory Standards Institute.^83^ In brief, few colonies were taken from the MHA overnight bacterial culture and inoculated into 0.9% saline solution, and vortexed to ensure the bacterial suspension was homogeneous. Bacterial suspensions were analyzed using a densitometer (DEN-1, BioSan, USA) and adjusted to 1 x 10^6^ colony forming units (CFU/mL) by diluting with CAMHB. Stocks of compounds were prepared at 20 mg/mL in dimethyl sulfoxide (DMSO, Sigma-Aldrich). The final primary screening concentration of compounds was 100 μg/mL once diluted into CAMHB, containing 1% DMSO final concentration. The assays were performed on sterile 96-well microtiter plates (Thermo Fisher Scientific). In each well, 100 μL of bacterial suspension was added into 100 μL of compound solution diluted into CAMHB. Plates were incubated at 37°C with 500 rpm agitation for 24 h (PST-60HL-4 Thermoshaker). Minimum inhibitory concentrations (MIC) were determined for selected compounds showing significant growth inhibitions (≥80%) as described above, but in a dose-response manner by testing eight 2-fold dilutions for each compound. Compound 1 was also tested on an extended panel of gram-positive bacterial species and antibiotic resistant strains (i.e., MRSA and VR Enterococci). Wells with media only were used as background controls. Positive controls were used to confirm correct performance of assays. Those consisted of wells containing ciprofloxacin (ICN Biomedical) for *S. aureus* and *E. faecalis* strains or linezolid (Sigma-Aldrich) for *E. faecium* strains, at MICs previously determined in our laboratory, i.e., *S. aureus*, MRSA and VR *E. faecalis* (0.5 μg/mL), *E. faecalis* (2 μg/mL); *E. faecium* and VR *E. faecium* (4 μg/mL). Experiments were conducted twice in triplicate. Combinatory assays were performed as described above but only one experiments in triplicate was performed. The concentration of 2 that was selected, 500μM, was based on findings described by Madeiro et al.^71^

#### *Caenorhabditis elegans* survival and motility assay

The *C. elegans* survival assay was performed in 96-well microtiter plates as previously described in,^17^ adapted from Solis and Petrascheck.^82^ Briefly, 5–18 age-synchronized L1 nematodes were transferred by pipetting to 96-well microtiter plates (Sarstedt) with 6 mg/mL OP50 in 90 μL LS-complete media, where they grow for 24 h at 25°C until all worms reach the L3 stage. 5-Fluorodeoxyuridine (FUdR; 0.12 mM final; Sigma-Aldrich) was added to sterilize the worms and to keep the population synchronized. The following day (day 0), test samples were added to the sterilized adult worm culture at a final concentration of 1% DMSO which is also used as a vehicle control. Reserpine 30 μM (Sigma-Aldrich) was used as positive control.^84^ Nematodes were oxygenized every three days and 3.8 μL OP50 (c = 100 mg/mL) was added on day 5 of adulthood to prevent starvation. Each sample/concentration was set-up in triplicate and all assays were performed thrice. To prevent evaporation, the outer wells were filled with 200 μL S-complete medium and plates were sealed with parafilm. Plates were stored at 25°C protected from light. The population was counted every 3 days with a microscope (Z1 Axio Observer, Zeiss) to keep track of the number of living worms per well. Percentage of survival on the 7^th^ day of adulthood was used as readout. Simultaneously, the motility of the worms was measured every second day with an infrared-based worm tracker (WMicrotracker One, Phylum TECH).

#### Larval development inhibition assay

The *C. elegans* development inhibition assay was performed as previously described,^14^ with some modifications: approximately 5–18 age-synchronized L1 larvae were transferred by pipetting to 96-well microtiter plates with 5 mg/mL OP50 in 90 μL S-complete medium. L1 larvae were treated with vehicle control (1% DMSO), PO01_05 (50 μg/mL), ostruthin (5–500 μM) without co-treatment, or ostruthin (5–500 μM) with co-treatment of (±)-verapamil HCL (Sigma-Aldrich), isoimperatorin or imperatorin at a final concentration of 50 μM. Plates were left in the dark at 25°C and after a time period of 52–55 h (vehicle control-treated larvae were developed to L4 or adult worms), the larvae/worms were differentiated according to their development. L1-L3 larvae were classified as inhibited, whereas L4 larvae and adult worms were classified as developed. Development was calculated in percentages as number of L4/adult worms divided by the number of total larvae/worms per well. Each sample/concentration was set-up in triplicate and all assays were performed three times.

### Quantitation and statistical analysis

#### UPLC-ELSD analysis

Chromatographic analyses were performed using a Waters Acquity UPLC system (H-class) equipped with a binary solvent manager, a sample manager, a column manager, a PDA detector, an ELSD, and a fraction collector. The UPLC system was further coupled to an Acquity QDa mass detector with an electrospray ionization source and an isocratic solvent manager as a make-up pump. A dereplication of the relevant microfractions (i.e., PO01_05 – PO01_10) in the positive ionization mode (*m*/*z* 100–1000) was performed using a make-up flow rate of 0.150 mL/min with 10 mM ammonium formate in a mixture of 95% H_2_O and 5% CH_3_OH. All 31 PO-E derived microfractions were chromatographed over a Waters Acquity UPLC BEH Phenyl column (1.7 μm, 2.1 × 100 mm) using a binary mobile phase system consisting of A) H_2_O and B) CH_3_CN. The gradient was from 13 to 98% B in 12 min followed by 5 min re-equilibration. Method in detail: 13% B for 0.5 min, 13–18% B in 0.5 min, 18–45% B in 1 min, isocratic 45% B for 1.7 min, 45–73% B in 2.8 min, 73–98% B in 0.3 min, isocratic 98% B for 5 min, 98–13% B in 0.1 min, isocratic 13% B for 0.1 min; Conditions: temperature, 40°C; flow rate, 0.300 mL/min; injection volume, 1 μL. Detection of compounds using PDA and ELSD. PDA conditions: 210 nm and full range spectra 192–400 nm.

#### NMR analysis and statistical correlation with bioactivity data

NMR experiments were performed on a Bruker Avance 500 NMR spectrometer (UltraShield; Bruker, Billerica, MA) with a 5 mm probe (TCI Prodigy CryoProbe, 5 mm, triple resonance inverse detection probe head) with z-axis gradients and automatic tuning and matching accessory (Bruker BioSpin). The resonance frequency for ^1^H NMR was 500.13 MHz and for ^13^C NMR 125.75 MHz. Standard 1D experiments were used as supplied by the manufacturer. Ultrahigh-gradient grade solvents from Merck (Darmstadt, Germany) and deuterated solvents from Deutero GmbH (Kastellaun, Germany) were used. Spectral alignment, baseline correction and detection of structural features of the active compounds prior to isolation was done via heterocovariance analysis (HetCA) and statistical total correlation spectroscopy (STOCSY) analysis as previously described.^36^^,^^40^ Briefly, ^1^H NMR spectra of relevant fraction packages were bucketed (covered range: *δ*_H_ 0.5–10; bucket width: 0.0005 ppm), the intensity of ^1^H NMR resonances of each bucket was calculated and served as variables for the subsequent analysis. Covariance between the two variables (i) ^1^H NMR resonance intensity and (ii) percentage of *S. aureus* growth inhibition (at 100 μg/mL) or *C. elegans* survival (at 50 μg/mL) were calculated and the correlation coefficient was calculated for color coding. HetCA analyses were applied for spectra of selected sets of microfractions that showed a distinct variation in bioactivity and concentration of contained secondary metabolites. ^1^H NMR pseudo-spectra were color coded according to their respective correlation coefficients which allowed for a straightforward identification of ^1^H NMR resonances which were either positively (red) or negatively (blue) correlated with *S. aureus* growth inhibition or *C. elegans* survival rates. HetCA and STOCSY analyses were performed using the software MATrix LABoratory (MATLAB).

#### Antibacterial activity

Absorbance values measured at 600 nm using the MultiskanGO plate reader (Thermo Fisher Scientific) were used for evaluating the antibacterial effects, by comparing to untreated controls, and expressed as percentage of growth inhibition. MIC values were defined as the lowest compound concentration at which bacterial growth was inhibited by 90% compared to maximum bacterial growth control after 24 h of incubation at 37°C.

#### *C. elegans* assays

Raw data of the survival assay was recorded with MS Excel 2019 to keep track of living/dead population per well. Survival curves for each plate were determined based on the percentage of living nematodes per well plotted versus time. At least 3 wells per trial were used for each condition. The survival rate (%) on the 7^th^ day of the experiment was used. The deviation of lifespan in comparison to the vehicle control is given as increase/decrease in percentage. For better visualization, all results were depicted as bar charts (GraphPad Prism 6) and data values were reported as the mean ± SD. To determine whether the differences between control and treated groups were statistically significant, an ANOVA (analysis of variance) with Dunnett’s post-test was performed. Significant activity is based on p < 0.05. For the larval-development inhibition assay, logarithm of doses against percentage of developed larvae were plotted and the IC_50_ value of each treatment was calculated from the mean of three replicates (GraphPad Prism 6).

## Data Availability

• All data reported in this paper will be shared by the lead contact upon request. • This paper does not report original code. • Any additional information required to reanalyze the data reported in this paper is available from the lead contact upon request.

## Supplemental information

Document S1. Figure S1 and Tables 6

Table S1. E. coli ATCC 25922 and S. aureus ATCC 29213 growth inhibition (in percentage) obtained during the initial screening of 139 herbal and 19 fungal extracts tested at 100 μg/mL, related to the STAR Methods (experimental model and study participant details, extracts) LLE, lead-like enhanced extracts; E, ethanolic extracts; D, dichloromethane extracts; M, methanol; ASE, accelerated solvent extraction; SFE, supercritical-fluid extraction. ∗Ciprofloxacin at 0.02 μg/mL and 0.5 μg/mL was used as positive control for *E. coli* and *S. aureus*, respectively. Experiment was performed once in triplicate.
