# Supplementary material for: Biochemometry identifies ostruthin as pluripotent antimicrobial and anthelmintic agent from masterwort
Source: iScience. 2023 Aug 3;26(9):107523. doi: 10.1016/j.isci.2023.107523 (PMC10457539; doi:10.1016/j.isci.2023.107523)
Supplement: Document S1. Figure S1 and Tables 6 [file mmc1.pdf]

iScience, Volume *26*

Supplemental information

Biochemometry identifies ostruthin as pluripotent antimicrobial and anthelmintic agent from masterwort

Julia Zwirchmayr, Cristina D. Cruz, Ulrike Grienke, Päivi Tammela, and Judith M. Rollinger

SUPPLEMENTARY INFORMATION

Table S2. Results from the screening of the 31 microfractions *from PO-E* (PO01_01 – 31), related to Figure 1. A.) against *S. aureus* (test conc. 100µg/mL), and B.) against *C. elegans* (test conc. 50µg/mL).

Sample		Inhibition of bacterial growth (%)		Survival rates of the nematodes at the 7th day of treatment (%)		
		S. aureus ATCC 29213		*C. elegans* N2		
		Average	SD	Mean	SD	*N*
DMSO 1%	vehicle control	-	-	67.35	4.05	259
Reserpine 30 µM	positive control	-	-	80.61	4.31	265
Ciprofloxacin 0.5 µg/mL	positive control	99.6	0.1	-	-	-
PO-E	extract	77.7	0.9	27.17	5.44	291
PO01_01	microfraction	19.2	2.8	57.39	8.99	112
PO01_02	microfraction	27.4	1.5	62.05	10.43	91
PO01_03	microfraction	21.6	8.0	69.52	12.86	109
PO01_04	microfraction	35.1	1.5	63.71	2.57	114
**PO01_05**	**microfraction**	**85.5**	**0.9**	**44.02**	**4.77**	**101**
**PO01_06**	**microfraction**	**116.7**	**1.2**	**65.11**	**8.91**	**91**
**PO01_07**	**microfraction**	**105.1**	**1.3**	**67.87**	**3.90**	**122**
**PO01_08**	**microfraction**	**83.9**	**0.9**	**50.68**	**3.13**	**100**
**PO01_09**	**microfraction**	**90.3**	**1.5**	**57.66**	**4.53**	**114**
PO01_10	microfraction	0	5.6	76.24	5.53	99
PO01_11	microfraction	0	7.7	72.05	16.09	99
PO01_12	microfraction	8.3	7.9	66.56	5.03	119
PO01_13	microfraction	7.4	4.1	65.31	9.37	116
PO01_14	microfraction	0	3.2	67.13	16.05	92
PO01_15	microfraction	35.4	3.5	67.08	13.60	102
PO01_16*	microfraction	21.8	10.3	68.18	8.47	138
PO01_17	microfraction	22.4	15.4	67.04	8.05	118
PO01_18	microfraction	21.7	10.9	67.19	9.93	114
PO01_19	microfraction	29.1	10.0	65.36	7.74	102
PO01_20	microfraction	32.6	11.2	63.09	8.89	103
PO01_21	microfraction	19.9	7.7	69.53	12.81	106
PO01_22	microfraction	12.5	5.0	77.39	8.32	104
PO01_23	microfraction	24.2	8.4	65.20	1.69	111
PO01_24	microfraction	20.5	4.4	55.12	5.40	116
PO01_25	microfraction	11.7	2.6	66.08	8.56	115
PO01_26	microfraction	15.7	9.8	64.13	6.14	96
PO01_27	microfraction	24.8	5.0	58.33	15.37	121
PO01_28	microfraction	19.1	4.3	55.80	8.67	109
PO01_29	microfraction	43.4	7.8	58.83	19.71	108
PO01_30	microfraction	25.9	7.0	70.42	4.86	103
PO01_31	microfraction	27.8	5.6	71.30	12.95	94

*tested at 50 µg/mL due to availability. Bold font represents microfractions which significant bacterial growth inhibition. Experiment was performed in triplicates.
Data of the nematodes' mean survival rates were generated from three parallel experiments. *N* is the total number of worms per sample.

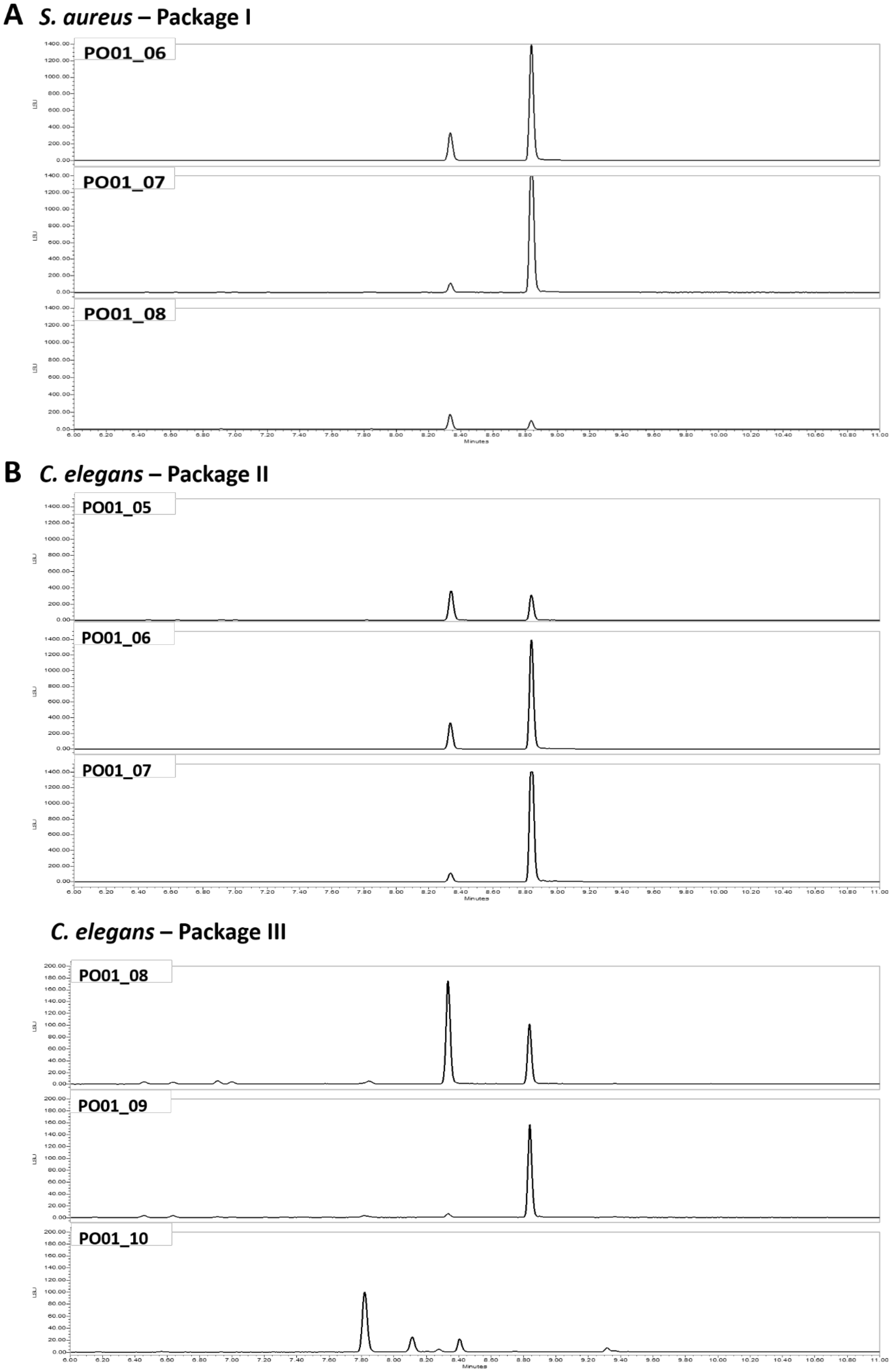

Figure S1. UPLC-ELSD Chromatograms of selected microfractions, related to Figure 1. A.) Package I (PO01_06 – PO01_08) to identify antibacterial compounds against *S. aureus*. B.) Package II (PO01_05 – PO01_07) and Package III (PO01_08 – PO01_10) used to identify anthelmintic constituents against *C. elegans*.

Table S3. Nematodes' survival analysis, related to Figure 5. A.) upon treatment with controls and **1** and **2** at concentrations ranging from 5 – 500 μM in adult nematodes; B.) treatment with different concentrations of a 1:1 mixture of **1** and **2** ranging from 500 – 5 μM.

**Sample**	**Concentration**	**Survival rates of the nematodes at the 7th day of treatment (%)**		
		**Mean**	**SD**	***N***
(**A**)				
**Control**	-	66.21	4.4	166
**Reserpine**	30 μM	77.6	6.8	188
**PO01_07**	50 μg/mL	60.43	3.0	56
**1**	500 μM	58.11	11.4	134
**1**	250 μM	60.76	5.9	142
**1**	100 μM	67.8	12.2	117
**1**	50 μM	66.57	9.7	139
**1**	25 μM	71.05	12.1	133
**1**	10 μM	61.4	10.1	131
**1**	5 μM	69.65	1.8	130
**PO01_05**	50 μg/mL	26.18	11.2	75
**2**	500 μM	69.77	7.1	148
**2**	250 μM	68.24	9.7	150
**2**	100 μM	70.76	0.8	133
**2**	50 μM	68.86	6.4	125
**2**	25 μM	70.82	12.8	86
**2**	10 μM	69.63	7.8	103
**2**	5 μM	67.85	8.1	108
(**B**)				
DMSO	1%	63.58	4.08	178
Reserpine	30 μM	75.99	2.88	168
PO01_05	50 μg/mL	22.29	3.01	163
**1** + **2** [1:1 mix]	500 μM	8.307	1.83	136
**1** + **2** [1:1 mix]	250 μM	18.73	3.16	169
**1** + **2** [1:1 mix]	100 μM	29.49	3.18	149
**1** + **2** [1:1 mix]	50 μM	43.73	7.26	143
**1** + **2** [1:1 mix]	25 μM	42.73	1.99	144
**1** + **2** [1:1 mix]	10 μM	60.83	5.11	107
**1** + **2** [1:1 mix]	5 μM	66.98	5.84	171

Data were generated from three parallel experiments. *N* is the total number of worms per sample.

Table S4. Locomotor activity of *C. elegans* upon treatment with controls (vehicle control, levamisole, PO01_05) **and samples, related to Figure 6.** A.) various concentrations of **1**, B.) various concentrations of **2**, and C.) various concentrations of a 1:1 mix of **1** and **2**.

(A)

**Sample**		**day 3**			**day 5**			**day 7**		
		**%-worm motility**			**%-worm motility**			**%-worm motility**		
		**Mean**	**SD**	**%-reduction**	**Mean**	**SD**	**%-reduction**	**Mean**	**SD**	**%-reduction**
**Control**	-	88.31	7.9	-	60.92	15.9	-	41.78	6.1	-
**Levamisole**	10 µM	10.59	3.7	88.01	5.33	3.3	91.25	0.48	0.5	98.85
**PO01_05**	50 µg/mL	16.60	9.8	81.20	13.91	14.9	77.16	1.84	1.2	95.59
**1**	500 µM	54.24	21.1	38.58	27.42	17.3	54.99	11.43	12.7	72.64
**1**	250 µM	51.48	19.2	41.71	19.53	4.9	67.95	12.62	1.8	69.79
**1**	100 µM	83.94	6.9	4.95	36.26	9.5	40.47	27.56	9.3	34.03
**1**	50 µM	66.42	29.7	24.79	50.57	13.8	16.99	39.77	9.1	4.82
**1**	25 µM	78.90	19.2	10.66	52.63	26.6	13.60	45.75	16.9	-9.49
**1**	10 µM	85.74	37.3	2.91	61.86	30.9	-1.54	55.92	5.0	-33.84
**1**	5 µM	49.64	17.3	43.79	35.28	6.4	42.09	32.20	20.4	22.94

(**B**)

**Sample**		**day 3**			**day 5**			**day 7**		
		**%-worm motility**			**%-worm motility**			**%-worm motility**		
		**Mean**	**SD**	**%-reduction**	**Mean**	**SD**	**%-reduction**	**Mean**	**SD**	**%-reduction**
**Control**	-	88.31	7.9	**-**	60.92	15.9	**-**	41.78	6.1	**-**
**Levamisole**	10 µM	10.59	3.7	**88.01**	5.33	3.3	**91.25**	0.48	0.5	**98.85**
**PO01_05**	50 µg/mL	16.60	9.8	**81.20**	13.91	14.9	**77.16**	1.84	1.2	**95.59**
**2**	500 µM	97.16	13.9	**-10.02**	48.29	3.6	**20.72**	43.52	16.4	**-4.16**
**2**	250 µM	86.13	51.8	**2.47**	49.65	24.8	**18.49**	53.01	9.2	**-26.88**
**2**	100 µM	85.95	24.3	**2.68**	55.64	19.6	**8.66**	41.63	21.4	**0.35**
**2**	50 µM	100.09	17.2	**-13.34**	60.67	31.2	**0.40**	47.53	22.1	**-13.75**
**2**	25 µM	93.42	7.1	**-5.78**	61.78	48.2	**-1.41**	59.79	17.1	**-43.10**
**2**	10 µM	61.31	13.7	**30.57**	53.53	21.7	**12.13**	33.84	13.1	**19.00**
**2**	5 µM	89.52	6.5	**-1.37**	59.49	24.8	**2.35**	40.15	15.0	**3.90**

(**C**)

**Sample**		**day 3**			**day 5**			**day 7**		
		**%-worm motility**			**%-worm motility**			**%-worm motility**		
		**Mean**	**SD**	**%-reduction**	**Mean**	**SD**	**%-reduction**	**Mean**	**SD**	**%-reduction**
**Control**	-	74.93	10.9	**-**	44.86	10.2	**-**	21.95	0.6	**-**
**Levamisole**	10 µM	16.55	9.7	**77.91**	2.48	3.4	**94.47**	1.21	0.7	**94.47**
**PO01_05**	50 µg/mL	30.42	1.1	**59.40**	8.64	6.0	**80.74**	4.43	2.3	**79.84**
**1+2** [1:1 mix]	500 µM	14.49	7.8	**80.67**	11.88	0.6	**73.53**	0.29	0.3	**98.69**
**1+2** [1:1 mix]	250 µM	35.95	3.2	**52.02**	6.05	1.1	**86.51**	1.92	2.5	**91.27**
**1+2** [1:1 mix]	100 µM	39.07	20.6	**47.86**	9.76	1.0	**78.24**	2.04	0.8	**90.71**
**1+2** [1:1 mix]	50 µM	50.87	18.4	**32.11**	28.48	27.9	**36.51**	10.04	0.9	**54.26**
**1+2** [1:1 mix]	25 µM	48.22	8.6	**35.65**	29.24	2.3	**34.83**	5.01	0.3	**77.16**
**1+2** [1:1 mix]	10 µM	46.23	6.6	**38.30**	33.30	16.5	**25.76**	18.74	13.1	**14.62**
**1+2** [1:1 mix]	5 µM	78.34	16.3	**-4.55**	43.63	16.2	**2.73**	14.65	6.8	**33.27**

The basal activity of the worms was measured on day 0 using an IR-based wormtracker (wMicro-tracker TM ONE); data were normalized to the basal activity. The locomotor activity (in percentage ± SD) was measured on the 3rd, 5th, and 7th of the treatment and the mean locomotor activity was calculated based on three parallel experiments.

Table S5. Results from the larval development inhibition assay upon treatment of L1 larvae with controls (vehicle control and PO01_05) **and samples, related to Figure 7.** A.) with **1** at various concentrations and **1** in presence of 50 μM **2**, **3** or **4**, respectively.

**Sample**		**% developed**			**% inhibition (compared to control)**
		**Mean**	**SD**	***N***	
Control	**-**	93.17	2.1	260	-
PO01_05	**50 μg/mL**	40.74	4.4	274	56.27
**1**	**500 μM**	5.66	2.6	171	93.93
**1**	**250 μM**	80.97	1.9	175	13.09
**1**	**100 μM**	82.40	3.4	164	11.56
**1**	**50 μM**	82.73	4.6	155	11.21
**1**	**25 μM**	86.01	3.3	159	7.68
**1**	**10 μM**	91.39	1.1	175	1.91
**1**	**5 μM**	90.17	2.5	162	3.22
**1** + **2** (50 μM)	**500 μM**	0.00	0.0	179	100.00
**1** + **2** (50 μM)	**250 μM**	44.89	2.0	157	51.82
**1** + **2** (50 μM)	**100 μM**	56.18	7.1	181	39.70
**1** + **2** (50 μM)	**50 μM**	63.73	6.0	147	31.60
**1** + **2** (50 μM)	**25 μM**	71.08	5.2	173	23.71
**1** + **2** (50 μM)	**10 μM**	77.19	6.6	169	17.15
**1** + **2** (50 μM)	**5 μM**	87.42	2.5	158	6.17
**1** + **3** (50 μM)	**500 μM**	0.00	0.0	156	100.00
**1** + **3** (50 μM)	**250 μM**	50.53	4.6	163	45.77
**1** + **3** (50 μM)	**100 μM**	60.08	4.4	138	35.52
**1** + **3** (50 μM)	**50 μM**	66.15	4.2	133	29.00
**1** + **3** (50 μM)	**25 μM**	69.77	4.1	153	25.12
**1** + **3** (50 μM)	**10 μM**	75.80	4.5	142	18.64
**1** + **3** (50 μM)	**5 μM**	79.02	10.8	156	15.19
**1** + **4** (50 μM)	**500 μM**	0.00	0.0	149	100.00
**1** + **4** (50 μM)	**250 μM**	38.26	1.2	177	58.94
**1** + **4** (50 μM)	**100 μM**	49.71	3.3	148	46.65
**1** + **4** (50 μM)	**50 μM**	61.86	4.7	168	33.61
**1** + **4** (50 μM)	**25 μM**	63.11	7.5	152	32.26
**1** + **4** (50 μM)	**10 μM**	73.18	5.6	173	21.46
**1** + **4** (50 μM)	**5 μM**	79.36	7.9	174	14.82

Percentages of development were calculated as fraction of L1-L3 developmental stages relative to L4 and adult worms. Percentages of the inhibition were calculated in comparison to the vehicle control-treated worms.

Table S6. ***S. aureus*** **ATCC 29213 growth inhibition (in percentage) in presence of 1 and in combination with 2, related to Table 2**. Compound **1** at 12.5 μM was used as positive control, respectively. Experiment was performed once in triplicate.

Compound (Concentration)	Growth inhibition (%)	SD
**1** (12.5 μM)	96.00	7.49
**1** (6.25 μM)	0	5.29
**1** (3.13 μM)	0	6.44
**2** (500 μM)*	0	0.94
**1** (3.13 μM) + **2** (500 μM)	0	3.60
